# Development and expert consensus validation of a technological resource to support the integration of palliative care from hospital to home: A Delphi study

**DOI:** 10.1017/S1478951526102156

**Published:** 2026-03-30

**Authors:** Sara Cruz, Carla Sílvia Fernandes, Bruno Magalhães

**Affiliations:** 1 Department of Nursing Sciences, Abel Salazar Institute of Biomedical Sciences (ICBAS), University of Porto, Porto, Portugal; 2 Oncology Nursing Research Unit IPO Porto Research Center (CI-IPOP), Portuguese Oncology Institute of Porto (IPO Porto), Porto, Portugal; 3 Porto Comprehensive Cancer Centre (Porto.CCC) & RISE@CI-IPOP (Health Research Network), Porto, Portugal; 4 Department of Nursing, School of Health, Polytechnic Institute of Viana do Castelo, Portugal; 5 Rise@health Research Unit, Porto, Portugal; 6 ADITGames Association, Póvoa do Varzim, Portugal; 7 School of Health, Department of Nursing, University of Trás-os-Montes and Alto Douro (UTAD), Vila Real, Portugal; 8 RISE@UTAD-Health Research Network, Faculty of Medicine, University of Porto, Porto, Portugal; 9 Clinical Academic Centre of Trás-os-Montes and Alto Douro (CACTMAD), Vila Real, Portugal

**Keywords:** Palliative care, digital health, Delphi technique, home care services, patient-centered care

## Abstract

**Objective:**

The effective integration of palliative care along the hospital–home trajectory remains a challenge, with digital technologies representing a promising strategy to improve continuity and coordination of care. This study aimed to validate, through expert consensus, the objectives, functionalities, clinical content, organizational requirements, and barriers and facilitators of a technological resource to support the integration of palliative care from hospital to home.

**Methods:**

A methodological consensus study using a modified Delphi technique was conducted over two rounds. A multidisciplinary panel of experts with experience in palliative care, digital health, and healthcare organization participated. In the first round, experts evaluated an initial set of items derived from the literature and clinical practice. Items were analyzed for consensus and, based on qualitative comments, linguistically refined. In the second round, experts reassessed the items to confirm consensus and evaluate the stability of responses. A 4-point Likert scale was used. Consensus was defined as ≥75% of responses indicating “Agree” or “Strongly agree,” with calculation of the Item Content Validity Index (I-CVI) and the coefficient of variation.

**Results:**

Thirty-three experts participated in both rounds, corresponding to a 100% retention rate. All items reached consensus in the first round and maintained consensus and high stability in the second round. Agreement levels were high across all domains, with I-CVI values ≥0.78 and coefficients of variation below 0.25, confirming the content validity of the final set of items. No items were excluded throughout the Delphi process.

**Significance of results:**

This study validated a comprehensive and structured set of essential components for the development of a technological resource to support the integration of palliative care from hospital to home. The high levels of consensus and stability achieved support the clinical and organizational relevance of the resource, providing a solid foundation for its development, implementation, and future evaluation.

## Introduction

The timely and continuous integration of palliative care along the care trajectory of people with advanced disease is widely recognized as a key determinant of care quality, particularly during the transition from hospital to home (World Health Organization 2020a; Adamidis et al. 2024). Despite international recommendations advocating continuity of care and coordination across levels of care, significant gaps persist in care coordination, communication between teams, and support for people in palliative situations and their caregivers in the home setting (Cruz et al. 2025, 2025a). These shortcomings may result in clinical insecurity, symptom exacerbation, inappropriate use of emergency services, and increased suffering for both people and families.

For many people in palliative situations, the home is the preferred place of care (Pinto et al. 2024). However, this option requires effective support structures, timely access to specialized professionals, and monitoring and communication mechanisms that ensure an adequate response to emerging needs (Isenberg et al. 2021; Liu et al. 2021; Cruz et al. 2025b). The lack of integrated follow-up systems after hospital discharge continues to represent a major challenge for healthcare systems, particularly with regard to early identification of clinical deterioration, shared decision-making, and continuous support for informal caregivers.

The development of digital health resources has been identified as a promising strategy to improve continuity of care, symptom monitoring, communication across levels of care, and the empowerment of people and families (Bienfait et al. 2020; Disalvo et al. 2021; Harding et al. 2021). In the context of palliative care, digital solutions can facilitate remote follow-up, access to clinical and educational information, and coordination between hospital teams, primary care, and community-based services (Pavic et al. 2020; Nwosu et al. 2022). However, the effective adoption of these solutions depends on their clinical, organizational, and ethical appropriateness, as well as their acceptance by healthcare professionals, people in palliative situations, and caregivers (Cox et al. 2018; Tan et al. 2024).

The literature indicates that many technological resources fail because they do not reflect the real needs of users or are not aligned with the organizational contexts in which they are implemented (Meier 2018; Nkhoma et al. 2021; Oelschlägel et al. 2021; Madaki et al. 2024). Therefore, involving experts with clinical, organizational, and scientific experience in the design and validation process of such resources is considered a key element to ensure their relevance, applicability, and sustainability.

The Delphi technique has been widely used in healthcare contexts for the development and validation of frameworks, competencies, training program, and complex interventions, enabling structured consensus among specialists (Nasa et al. 2021b; Furtado et al. 2024). In the context of digital palliative care, this methodology is particularly relevant, given the need to integrate clinical, organizational, technological, and person-centered perspectives in a field characterized by high complexity and ethical sensitivity.

This study forms part of the development of a technological resource to support the integration of palliative care from hospital to home and aims to validate, through expert consensus, the requirements, functionalities, clinical content, and organizational factors essential for its design and implementation. By using a two-round Delphi process, the study seeks to ensure that the proposed resource reflects a robust and consensual conceptual basis aligned with best practices in palliative care.

## Methods

### Study design

A methodological consensus study was conducted using a modified Delphi technique (Nasa et al. 2021a), developed over two successive rounds of online questionnaires. The Delphi technique is particularly suitable for the validation of complex health interventions, as it allows the achievement of structured consensus among experts while maintaining anonymity of responses and reducing the influence of hierarchical dynamics or individual dominance (Linstone and Turoff 1975; Keeney et al. 2001).

A modified Delphi approach was adopted because the process began with a predefined set of domains and items, rather than with a fully open initial round. These domains and items were grounded in a comprehensive program of prior research, including two systematic reviews and three primary phenomenological studies exploring the experiences and needs of community palliative care professionals, family caregivers, and people in palliative situations during the hospital-to-home transition. This empirical and theoretical foundation ensured that the proposed technological resource was anchored in real-world clinical practice and lived experience (Keeney et al. 2001; Fernandes and Magalhães 2024).

### Expert panel

The Delphi panel consisted of experts with recognized experience in the fields of palliative care, nursing, medicine, digital health, and the organization and management of healthcare services. Participants were selected using purposive sampling, a strategy recommended in Delphi studies to ensure diversity of perspectives and depth of expertise (Hasson et al. 2000).

Inclusion criteria comprised at least one of the following aspects: clinical experience in palliative care; academic or research activity in the field of palliative care and/or health technologies; involvement in innovation, organization, or care transition projects; or experience in the development, implementation, or evaluation of health interventions.

The relevant experience of the panel was described descriptively in order to document its adequacy in relation to the study objectives. Experts presented a mean of 16.9 years of professional practice and a mean of 6.4 years of experience in palliative care, indicating a panel with consolidated clinical, academic, and organizational experience, as well as diverse professional trajectories.

The number of participants per round was considered adequate, in accordance with methodological recommendations indicating that panels comprising between 15 and 35 experts are sufficient to achieve robust consensus (Keeney et al. 2001; Diamond et al. 2014).

### Delphi procedure

The Delphi process was conducted over two rounds, using electronic questionnaires administered via the Microsoft Forms^®^ platform.

### First Delphi round

The first round aimed to assess the relevance, clarity, and adequacy of the initial domains and items of the technological resource, which had been previously structured based on the scientific literature and earlier exploratory studies of the project. Experts were invited to rate each item and to provide qualitative comments or suggestions.

All items assessed in the first round reached consensus according to the predefined criteria, and no items were excluded or newly added. Experts’ suggestions resulted only in linguistic adjustments and conceptual clarifications, which were incorporated prior to the subsequent round.

### Second Delphi round

The second Delphi round had the following main objectives: to confirm the previously achieved consensus; to assess the stability of responses after the linguistic reformulation of items; and to definitively validate the domains, requirements, functionalities, and contents of the technological resource.

No new items were introduced in the second Delphi round, as all items proposed in the previous round had met the predefined consensus criterion (≥75% agreement). According to the methodological literature on the Delphi technique, introducing new items after consensus has been achieved may compromise comparability between rounds and unnecessarily prolong the process, shifting the focus away from confirmation and stabilization of responses. Thus, when the purpose of a subsequent round is to confirm the stability of experts’ opinions following linguistic adjustments or conceptual clarifications, it is recommended to retain the previously consensualized set of items without adding new elements (Hasson et al. 2000; Keeney et al. 2001). This methodological option allowed a rigorous assessment of response consistency and supported the closure of the Delphi process after the second round.

### Instrument and rating scale

The data collection instrument consisted of a structured questionnaire organized into thematic domains, integrating items related to clinical, educational, and supportive content; organizational and technological requirements; and barriers and facilitators to the implementation of the resource.

Each item was assessed using a 4-point Likert scale, without a neutral option, in order to encourage experts to take a clear position. The use of scales without a midpoint is frequently recommended in Delphi studies to reduce ambiguous responses and facilitate the interpretation of consensos (Diamond et al. 2014).

### Consensus and stability criteria

The definition of consensus and stability followed criteria widely used in Delphi studies in the health field: consensus was defined as ≥75% of responses rated as “Agree” or “Strongly agree” (Hasson et al. 2000); an Item Content Validity Index (I-CVI) ≥ 0.78, a value considered acceptable for panels with more than 15 experts (Polit and Beck 2006); and a coefficient of variation (CV) <0.25, indicative of low dispersion and good response stability (Keeney et al. 2001); Items showing greater statistical variability were retained with a note of attention and were not excluded, as they maintained overall consensus.

**Figure 1.**
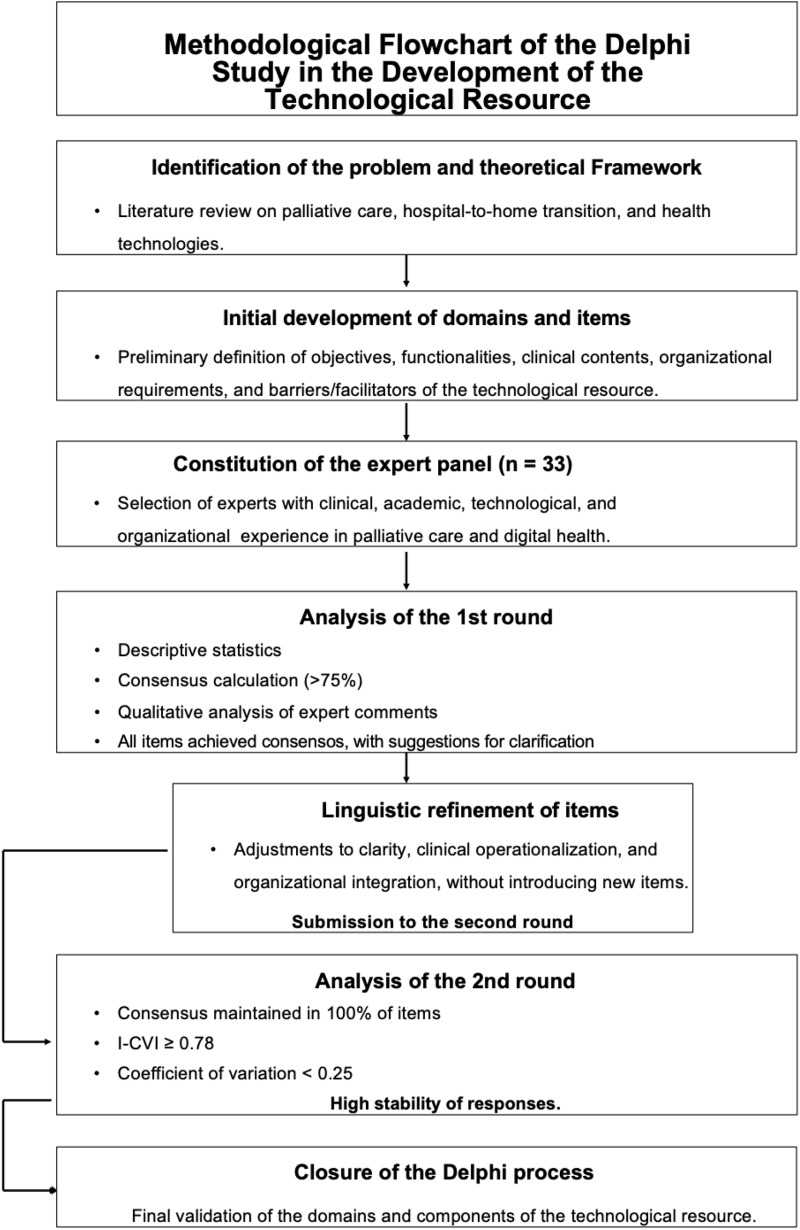
Methodological flowchart of the Delphi Study in the development of a technological resource to support the integration of palliative care.

The decision to prioritize both consensus and stability is aligned with contemporary approaches to the Delphi technique, which recognize response stability as a fundamental criterion for closing the process (Hasson et al. 2000; Nasa et al. 2021b). The overall methodological sequence of the Delphi process is presented in Figure 1 (see Fig. 1).

### Data analysis

Quantitative analysis included descriptive statistics for each item, namely, number of valid responses; mean and median; sample standard deviation; interquartile range; number and percentage of responses ≥4; calculation of the Item Content Validity Index (I-CVI); and the coefficient of variation.

Data were organized and analyzed descriptively, allowing comparison between rounds and assessment of the stability of consensus.

### Ethical considerations

The study was conducted in accordance with ethical principles for health research and with the Declaration of Helsinki. The study protocol was approved by the Ethics Committee of the University of Trás-os-Montes e Alto Douro (CE-UTAD), under approval Ref. Doc86-CE-UTAD-2025, issued on July 23, 2025. All participants received detailed information about the study objectives, procedures, confidentiality, and anonymity of responses, and voluntarily agreed to participate by providing informed consent.

## Results

### Characterization of the expert panel

Thirty-three experts participated in the first Delphi round, all of whom also completed the second round, corresponding to a 100% retention rate between rounds. The panel included professionals with clinical, academic, and/or research experience in palliative care, digital health, and healthcare organization, ensuring disciplinary diversity and complementary perspectives relevant to the objectives of the study (see Table 1).

**Table 1.** Sociodemographic characteristics of the Delphi experts

Rond 1 | Rond 2 *n* = 33		
	Frequency	%
**Sex**		
Male	16	48.5
Female	17	51.5
**Age**		
20–29	3	9.1
30–39	10	30.3
40–49	14	42.4
50–59	5	15.2
≥60	1	3.0
**Highest academic qualification**		
Bachelor’s degree	11	33.0
Master’s degree	18	54.5
Doctoral degree	4	12.1
**Professional category**		
Physician	8	24.2
Specialist Nurse	6	18.2
Digital Health Specialist	6	18.2
Nurse	5	15.2
Professor	4	12.1
Other (President of a professional association)	2	6.1
Nurse Manager	2	6.1
**Years of professional experience**		
0–5	5	15.2
6–10	8	24.2
11–20	8	24.2
21–30	9	27.3
>30	3	9.1
**Years of experience in palliative care**		
0	8	25.8
1–2	4	12.9
3–5	9	29.0
6–10	6	19.4
>10	4	12.9
**Professional setting**		
Hospital	12	36.4
Primary health care	11	33.3
Other (digital area)	6	18.2
Academic Institution	4	9.1

The experts had a mean age of 41.9 years (SD = 8.8; minimum = 25; maximum = 62), with greater representation in the 40–49 years (42.4%) and 30–39 years (30.3%) age groups. Regarding sex, the panel was balanced, with 51.5% female and 48.5% male participants.

The mean length of professional practice was 16.9 years (SD = 10.2), ranging from 0 to 41 years, with the majority of experts having more than 10 years of professional experience (60.6%), reflecting a panel with consolidated experience across different practice settings.

With respect to specific experience in palliative care, participants reported a mean of 6.4 years (SD = 7.7), with values ranging from 0 to 32 years. Notably, 29.0% of the experts had between 3 and 5 years of experience in palliative care, while 19.4% had between 6 and 10 years. A proportion of participants reported no direct clinical experience in this field (25.8%). This composition was intentional, allowing the integration of clinical, academic, technological, and organizational contributions into the development and validation of the technological resource.

### Results of the first Delphi round

In the first Delphi round, the experts evaluated an initial set of items related to the objectives, functionalities, clinical contents, organizational requirements of the technological resource, and barriers and facilitators to its implementation.

All items reached prior consensus (≥75% agreement), and therefore no items were excluded. The qualitative comments provided by the experts allowed the identification of needs for linguistic clarification, strengthening of clinical operationalization, and greater explicitness regarding aspects related to active follow-up, clinical response, and organizational integration.

Given that the aim of the first round was the structuring and initial validation of the domains and components of the technological resource, and considering that all items achieved consensus, the results are presented in a synthetic manner by domain. Detailed item-level analysis was reserved for the second Delphi round, which focused on confirming consensus and assessing the stability of responses following the linguistic refinement of the items (see Table 2).

**Table 2.** Results of the first Delphi round (summary). Summary of the levels of consensus achieved in the first Delphi round by domain. All items met the consensus criterion (≥75% agreement). Qualitative comments from experts highlighted the need for clarification and linguistic refinement; therefore, items were reformulated and submitted to a second round to confirm consensus and assess the stability of responses

Domain	No. of items	% Agreement	I-CVI	Decision
Objectives of the technological resource	5	≥ 90%	0.94–1.00	Retained
Functionalities of the technological resource	9	≥ 90%	0.91–1.00	Retained
Clinical, educational, and supportive contents	7	≥ 97%	0.97–1.00	Retained
Organizational and technological requirements	9	≥ 94%	0.94–1.00	Retained
Barriers and facilitators to implementation	10	≥ 90%	0.90–1.00	Retained

Based on these contributions, the items were linguistically refined while preserving their conceptual content and were subsequently submitted to a second Delphi round to confirm consensus and evaluate response stability.

### Results of the second Delphi round

The second Delphi round aimed to confirm the previously achieved consensus and to assess the stability of responses following the linguistic refinement of the items. Experts reassessed the items using a 4-point Likert scale (see Table 3).

**Table 3.** Results of the second Delphi round. Results by item and domain, including mean score, percentage agreement (≥Agree), I-CVI, and final decision. 4-point Likert scale (1 = Strongly disagree; 4 = Strongly agree)

Item	Domain	Mean	% Agreement	I-CVI	Decision
The resource should ensure continuity of care…	Objectives	3.97	**100%**	1.00	Retain
The resource should support a safe transition…	Objectives	3.97	**100%**	1.00	Retain
The resource should ensure bidirectional communication…	Objectives	3.91	**100%**	1.00	Retain
The resource should support clinical decision-making with alerts…	Objectives	3.76	**94%**	0.94%	Retain
The resource should be person- and informal caregiver-centered…	Objectives	3.97	**100%**	1.00	Retain
Symptom recording (text/image)	Functionalities	3.97	**100%**	1.00	Retain
Graduated automatic alerts	Functionalities	3.91	**100%**	1.00	Retain
Simple and intuitive graphs	Functionalities	3.97	**100%**	1.00	Retain
Rapid contact button	Functionalities	3.70	**91%**	0.91	Retain
Appointment scheduling/contact requests	Functionalities	3.52	**85%**	0.85	Retain
Multimedia educational library	Functionalities	3.88	**100%**	1.00	Retain
Validated scales (e.g., ESAS)	Functionalities	3.79	**94%**	0.94	Retain
Regular recording without user burden	Functionalities	3.94	**100%**	1.00	Retain
Secure messaging channel	Functionalities	3.70	**91%**	0.91	Retain
Management of prevalent symptoms	Clinical contents	3.94	**100%**	1.00	Retain
Videos and visual materials on procedures	Clinical contents	3.85	**100%**	1.00	Retain
Practical guidance on nutrition, comfort, and hygiene	Clinical contents	3.82	**94%**	0.94	Retain
Accessible explanations of the therapeutic plan	Clinical contents	3.70	**91%**	0.91	Retain
Support for emotional management and anticipatory grief	Clinical contents	3.61	**88%**	0.88	Retain
Guidance during crises/exacerbations, who to contact	Clinical contents	3.91	**100%**	1.00	Retain
Health literacy–adapted content	Clinical contents	3.94	**100%**	1.00	Retain
Compatibility with iOS and Android	Organizational requirements	3.94	**100%**	1.00	Retain
Collection of active and passive data	Organizational requirements	3.85	**100%**	1.00	Retain
Integration with electronic health records	Organizational requirements	3.97	**100%**	1.00	Retain
Training for healthcare teams	Organizational requirements	3.79	**94%**	0.94	Retain
Based on a theoretical model	Organizational requirements	3.70	**91%**	0.91	Retain
Accessible on mobile devices	Organizational requirements	3.91	**100%**	1.00	Retain
Functionality with low connectivity	Organizational requirements	3.85	**100%**	1.00	Retain
Robust data security and protection	Organizational requirements	3.97	**100%**	1.00	Retain
Co-construction with users and professionals	Organizational requirements	3.91	**100%**	1.00	Retain
Lack of interoperability compromises continuity	Barriers/facilitators	3.94	**100%**	1.00	Retain
Co-construction is essential for adoption	Barriers/facilitators	3.85	**100%**	1.00	Retain
Lack of funding affects sustainability	Barriers/facilitators	3.91	**100%**	1.00	Retain
Lack of clinical response reduces usefulness	Barriers/facilitators	3.70	**91%**	0.91	Retain
Low digital literacy as a barrier	Barriers/facilitators	3.64	**88%**	0.88	Retain
Team resistance may compromise implementation	Barriers/facilitators	3.55	**85%**	0.85	Retain
Dedicated technical support is an essential facilitator	Barriers/facilitators	3.82	**94%**	0.94	Retain
Collaboration across care levels is fundamental	Barriers/facilitators	3.88	**100%**	1.00	Retain
Simplicity and usability are key determinants	Barriers/facilitators	3.94	**100%**	1.00	Retain
Trust in data security determines uptake	Barriers/facilitators	3.97	100%	1.00	Retain

### Domain 1 – Objectives of the technological resource

All items related to the objectives of the technological resource maintained high levels of agreement. The percentage of “Agree” or “Strongly agree” responses exceeded 90% across all items, with I-CVI values ranging between 0.94 and 1.00 and coefficients of variation below 0.20, indicating strong consensus and high stability.

These findings confirm the importance attributed by experts to the role of the technological resource in continuity of care, hospital-to-home transition, bidirectional communication, and person- and caregiver-centered care.

### Domain 2 – Functionalities of the technological resource

Within the domain of functionalities, all items maintained consensus ≥75%, with most showing agreement rates above 90%. Functionalities related to symptom recording and monitoring, clinical alerts, rapid contact with the care team, secure messaging, and educational content were particularly valued.

Some items showed slightly higher coefficients of variation, reflecting greater heterogeneity in responses; however, this did not compromise overall consensus. These items were retained with a note of attention, particularly regarding the need to ensure timely clinical response and to avoid user burden.

### Domain 3 – Clinical, educational, and supportive contents

Items related to clinical, educational, and supportive contents demonstrated very high consensus, with agreement rates close to or equal to 100%. Experts highlighted the importance of content addressing symptom control, crisis management, emotional support for caregivers, and adaptation to health literacy levels, reinforcing the need for a clear, accessible, and clinically validated approach.

### Domain 4 – Organizational and technological requirements

In this domain, all items maintained both consensus and stability. Integration with existing information systems, data protection, team training, and co-construction with users and professionals were considered essential requirements for the implementation and sustainability of the technological resource.

### Domain 5 – Barriers and facilitators to implementation

Experts demonstrated high consensus regarding key barriers (e.g., interoperability, digital literacy, lack of timely clinical response) and facilitators (e.g., technical support, usability, inter-institutional collaboration). These findings reinforce the need for an integrated approach that considers not only technological design but also organizational and human contexts of implementation.

### Stability of responses and closure of the Delphi process

Analysis of the second round demonstrated high stability of responses, with coefficients of variation below 0.25 for all items and maintenance of consensus in 100% of items. According to methodological literature on the Delphi technique, these results support closure of the process after the second round, confirming validation of the final set of domains and items of the technological resource.

## Discussion

The present study aimed to validate, through expert consensus, the requirements, functionalities, clinical content, and organizational factors essential to the development of a technological resource to support the integration of palliative care from hospital to home. Using a modified two-round Delphi technique, high levels of consensus and response stability were achieved across all evaluated domains, enabling the final validation of the proposed set of items.

### Consensus and stability in the Delphi process

The results of the second Delphi round confirmed not only the maintenance of the previously achieved consensus but also the high stability of experts’ opinions, as reflected by low coefficients of variation and high I-CVI values. According to methodological literature, the simultaneous achievement of consensus and stability constitutes a robust criterion for concluding a Delphi process. This outcome is frequently observed after two rounds, particularly when items are derived from literature reviews and refined based on expert input (Hasson et al. 2000; Keeney et al. 2001).

The absence of item exclusion across both rounds suggests that the identified domains adequately reflect the needs perceived by experts in the context of palliative care integration, thereby reinforcing the content validity of the proposed technological resource.

### Objectives of the technological resource

The high level of consensus obtained for items related to the objectives of the technological resource highlights the importance attributed by experts to continuity of care, a safe and humanized transition from hospital to home, and effective communication between healthcare professionals, people receiving palliative care, and informal caregivers. These findings are aligned with international recommendations advocating for the early and continuous integration of palliative care, as well as the need for care models that overcome fragmentation across levels of care (Killackey et al. 2020; World Health Organization 2020b).

The consistent emphasis on the centrality of people receiving palliative care and their informal caregivers reinforces the relevance of person- and family-centered approaches, particularly in home-based contexts, where autonomy, comfort, and continuous support play a central role.

### Functionalities and remote monitoring

Within the domain of functionalities, the high consensus surrounding symptom recording and monitoring, clinical alerts, and bidirectional communication with palliative care teams confirms the potential of digital technologies to support clinical decision-making and timely responses to the needs of people receiving palliative care. These findings corroborate previous studies identifying remote monitoring and structured communication as key components of effective digital interventions in palliative and end-of-life care (Pavic et al. 2020; Disalvo et al. 2021; Harding et al. 2021).

However, some items showed greater variability in responses, reflecting experts’ concerns regarding the need to ensure adequate clinical responsiveness and to avoid overburdening users and care teams. Although this variability did not compromise overall consensus, it underscores the importance of careful implementation, with clearly defined response pathways and clinical responsibilities.

### Clinical, educational, and supportive content

Items related to clinical, educational, and supportive content demonstrated particularly high levels of agreement, highlighting the relevance of content focused on symptom control, crisis management, emotional support, and anticipatory grief. These findings reinforce the need to integrate educational and emotional support components into technological resources aimed at people receiving palliative care and their informal caregivers, extending beyond purely clinical monitoring.

The adaptation of content to users’ levels of health literacy was also highly valued, in line with evidence demonstrating that digital interventions are only effective when they are accessible, understandable, and culturally appropriate (Nittas et al. 2024; Wamala Andersson and Gonzalez 2025).

### Organizational requirements and implementation factors

The consensus achieved regarding organizational and technological requirements indicates that the success of a digital resource in palliative care is strongly dependent on the organizational context in which it is implemented. Integration with existing information systems, team training, data protection, and co-construction with users and professionals were considered essential elements to ensure adoption, sustainability, and safety.

These findings are consistent with literature highlighting that failures in interoperability, lack of training, and insufficient professional engagement are common barriers to the implementation of digital health technologies, regardless of their technical quality (Borges Do Nascimento et al. 2023; Avais et al. 2025).

### Barriers and facilitators to adoption

Within the domain of barriers and facilitators, experts identified critical factors that may influence the adoption of the technological resource, including users’ digital literacy, the availability of technical support, and timely clinical response. The simultaneous identification of barriers and facilitators reinforces the importance of a systemic approach to implementation, which considers not only the design of the technological resource but also the available human, financial, and organizational resources.

### Implications for practice and future research

The findings of this study provide a structured and validated foundation for the development of a technological resource to support the integration of palliative care from hospital to home. Validation through expert consensus helps to reduce the risk of developing solutions that are misaligned with real clinical and organizational needs.

Future research should focus on evaluating the acceptability, usability, and clinical impact of the resource in real-world settings, as well as analyzing its effects on the experiences of people receiving palliative care, informal caregivers, and healthcare teams.

## Conclusion

This study enabled the validation, through expert consensus, of a structured set of objectives, functionalities, clinical content, and organizational requirements for the development of a technological resource to support the integration of palliative care from hospital to home. The application of a modified two-round Delphi technique revealed high levels of consensus and response stability across all evaluated domains, supporting the content validity of the proposed resource.

The findings highlight the importance of digital solutions that promote continuity of care, effective communication across levels of care, and person-centered support for people receiving palliative care and their informal caregivers. Validation of these components by a multidisciplinary panel of experts reinforces the clinical and organizational relevance of the technological resource, providing a solid foundation for its development, implementation, and future evaluation in real-world contexts.

### Study limitations

Despite its methodological rigor, this study has some limitations. The Delphi technique is based on expert opinion and does not allow for direct inference regarding the clinical impact of the technological resource. Additionally, although the panel included professionals with diverse experience, the findings reflect perceptions from a specific context and may not be fully generalizable to other healthcare systems.

Future studies should therefore evaluate the acceptability, usability, and effectiveness of the resource among people receiving palliative care, informal caregivers, and healthcare teams in real-world practice settings.
